# Efficacy and cost-effectiveness of an experimental short-term inpatient Dialectical Behavior Therapy (DBT) program: study protocol for a randomized controlled trial

**DOI:** 10.1186/1745-6215-15-152

**Published:** 2014-05-01

**Authors:** Louisa M C van den Bosch, Roland Sinnaeve, Leona Hakkaart-van Roijen, Eric F van Furth

**Affiliations:** 1 Center for Personality Disorders Jelgersma, Postbox 750, 2300 Leiden, AT, the Netherlands; 2 Institute of Health Policy and Management (iBMG) and institute for Medical Technology Assessment (iMTA), Erasmus University Rotterdam, Postbus 1738, 3000 Rotterdam, DR, the Netherlands; 3 Department of Psychiatry, Leiden University Medical Center, Postbox 9600, 2300 Leiden, RC, the Netherlands

**Keywords:** Borderline Personality Disorder, Suicidal behavior, Self-harming behavior, Dialectical Behavior Therapy, Inpatient treatment

## Abstract

**Background:**

Borderline Personality Disorder (BPD) is a serious psychiatric condition associated with substantial mortality, burden and public health costs. DBT is the treatment model with the largest number of published research articles showing effectiveness. However, some patients are not sufficiently engaged in outpatient treatment while presenting severe parasuicidal behavior, making hospitalization necessary. The Center for Personality Disorders Jelgersma developed an intensive 12-week inpatient DBT program that (i) rapidly reduces core borderline symptoms like suicidal behavior, (ii) minimizes the negative effects of an inpatient setting, and (iii) enhances compliance with outpatient treatment. We evaluate the (cost-) effectiveness of this experimental program.

**Methods/design:**

Seventy patients, aged 18 to 45 years with a primary diagnosis of BPD, showing a chronic pattern of parasuicidal gestures and/or reporting high degrees of severity of other borderline symptoms, are randomly allocated to the control and intervention groups. Subjects in the control group receive standard outpatient DBT, provided in one of three regular mental health settings in GGZ Rivierduinen. Subjects in the intervention group receive 12 weeks of intensified inpatient DBT plus six months of standard DBT, provided in the Center for Personality Disorders Jelgersma. The primary outcome is the number of suicide attempts/self-harming acts. Secondary outcomes are severity of other borderline complaints, quality of life, general psychopathological symptoms and health care utilization and productivity costs. Data are gathered using a prospective, two (group: intervention and control) by five (time of measurement) repeated measures factorial design.

Participants will complete three-monthly outcome assessments in the course of therapy: at baseline, and 12, 24, 36 and 52 weeks after the start of the treatment. The period of recruitment started in March 2012 and the study will end in December 2014.

**Discussion:**

Highly suicidal outpatient patients can pose a dilemma for mental health care professionals. Although hospitalization seems inevitable under some circumstances, it has proven to be harmful in its own right. This paper outlines the background and methods of a randomized trial evaluating the possible surplus value of a short-term inpatient DBT program.

## Background

Borderline Personality Disorder (BPD) is a serious and prevalent psychiatric condition characterized by affective instability, impulsivity, and significant deficits in the capacity to work and maintain meaningful relationships. Patients with BPD struggle with a profound fear of abandonment, identity disturbances, and paranoid ideations. They are at risk for suicide and repetitive self-destructive behaviors. BPD patients show a completed suicide rate that is 50 times greater than that in the general population [1,2]. The short- to medium-term outcome of BPD is poor. There is some evidence that the long-term follow-up course is more favorable, with remission rates of about 88% within ten years [3]. However, ‘remission’ means that diagnostic criteria are not fulfilled. Affective symptoms reflecting areas of dysphoria, such as chronic feelings of emptiness, intense anger or profound abandonment, largely remain [4].

In the general population, the prevalence of BPD varies from 0.4 to 1.8%, with a pooled rate of 1.1% [5]. The lifetime prevalence of BPD was found to be 5.9% among a representative sample of the adult population of the United States [6,7]. In the Netherlands, the number of borderline patients is estimated at 100,000 [8]. In clinical samples, BPD is usually the most common personality disorder. In outpatient samples, rates of 9.3 to 18% have been reported, with a pooled rate of 11.9% [5]. Studies of psychiatric inpatient populations have reported rates of BPD at about 40% [9].

DBT is the BPD treatment model for BPD with the largest number of evidence-based published research articles on effectiveness (13 Random Controled Trials (RCT) versus 2 RCTs of next evidence treatment model off the rank). The American Psychological Association, Society of Clinical Psychology (APA) has cited DBT as one of the well-established, empirically supported, treatments for BPD that has strong research support [10]. The UK NICE guidelines recognize that, more than any other therapy, there is some evidence that DBT is effective in reducing suicide attempts and self-harm, anger, aggression and depression in patients with BPD [11]. This is also postulated in the Dutch guidelines [12].

Yet, standard DBT is lengthy and thus expensive. Moreover, some BPD patients are not sufficiently engaged in outpatient treatment and/or experience periodic exacerbations of severe self-injurious behavior making hospitalization necessary. A recent meta-analysis showed that psychotherapy for BPD yielded an overall drop-out rate of 25% [13]. Previous research also demonstrated that BPD patients are hospitalized and readmitted more than those with other disorders [14,15]. This is troublesome, since dropping out of treatment, as well as a pattern of inpatient treatments, adds to the suicide risk [16,17]. These figures frequently pose a dilemma for mental health care professionals treating highly suicidal patients in an outpatient setting. Hospitalization seems inevitable under some circumstances, yet has proven harmful in its own right. It is concluded that there is an urgent need to develop and test short-term inpatient treatment programs that (i) rapidly reduce core borderline symptoms like suicidal behavior, (ii) minimize the negative effects of an inpatient setting, and (iii) enhance compliance with outpatient treatment.

Following the publication of the SCEPTRE research results [18], and a growing number of inpatient DBT programs [19-22], the concept of inpatient treatment seems to be gaining interest. There is sufficient evidence that inpatient DBT leads to symptom reduction and that treatment gains are maintained after discharge for adult and adolescent BPD patients [23,24]. However, firm conclusions about the efficacy of inpatient DBT cannot be drawn. The effect of inpatient DBT has not been investigated in RCTs or compared to the effect of outpatient DBT. Moreover, no data on the cost-effectiveness of inpatient DBT are available.

The Center of Personality Disorders Jelgersma (GGZ Rivierduinen Leiden, Netherlands) developed an intensive 12-week inpatient DBT program that (i) rapidly reduces core borderline symptoms like suicidal behavior, (ii) minimizes negative effects of an inpatient setting, and (iii) enhances compliance with outpatient treatment. We evaluated the (cost-) effectiveness of this experimental program.

## Methods/design

### Study design

**Figure 1:**
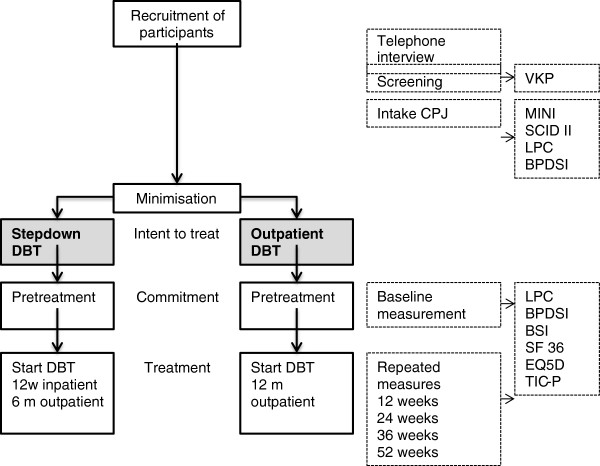
**Study design.** Procedures in a study comparing experimental short-term inpatient DBT program and standard outpatient DBT.

This is a prospective randomized controlled trial. It is a two (group: intervention and control) by five (time: pretreatment, 12 weeks, 24 weeks, 36 weeks, 52) repeated measures factorial design. The overall study design is illustrated in Figure 1.

### Participants

Participants will consist of seventy patients, aged 18 to 45 years with a primary diagnosis of Borderline Personality Disorder, according to the *Diagnostic and Statistical Manual of Mental Disorders* (DSM-IV), showing a chronic pattern of parasuicidal gestures and/or reporting high degrees of severity of other borderline symptoms. It was decided that Jelgersma Center for Personality Disorders in Oegstgeest, Regional Psychiatric Center GGZ Rijnstreek, Regional Psychiatric Center GGZ Leiden and Regional Psychiatric Center GGZ Midden-Holland, constituting all participating centers of GGZ Rivierduinen, would implement a central recruiting and diagnostic facility in order to recruit patients efficiently and uniformly. All referrals for initial assessment in this facility were considered, given that patients contact the researcher themselves and of their own free will. No restriction is made in terms of the referral source. Only hard drug abuse that requires inpatient detoxification and a forced treatment framework were refused. We predict that the majority of referrals will come from the Jelgersma Center for Personality Disorders, since this center has a nationwide reputation.

### Inclusion criteria

1) Aged between 18 and 45 years

2) Independently contacted the researcher or coached do to so

3) Fulfill the DSM-IV-TR criteria for BPD. Presence of BPD is according to semi-structured interview SCID-II, administered by a trained professional (psychologist, supervised by a project leader)

4) Show a severe level of borderline symptomatology (>24 on the Borderline Severity Index)

5) Parasuicidal behavior in the last month preceding baseline measurement and/or a profound level of borderline symptoms (>30 on the Borderline Severity Index)

6) Sufficient command of the Dutch language

7) Within travelling distance from Leiden

8) Understands and agrees with randomization procedure

### Exclusion criteria

1) IQ < 80

2) Chronic psychotic condition

3) Bipolar disorder

4) Hard drug abuse that requires inpatient detoxification

5) Forced treatment framework

6) Started DBT in the year preceding intake

7) Repeatedly fails to return the screening instrument or does not attend for a thorough examination

8) Disagrees with outcome of randomization

### Study conditions

A) Intensified adapted DBT program plus six months standard outpatient DBT

Twelve-week inpatient DBT was developed in the Jelgersma Center for Personality Disorders. This treatment unit provides accommodation for nine patients. Patients are admitted five days a week. Staff is only present in the daytime. During the weekends the patients stay at home. The therapy [16] consists of DBT skills training [25], individual psychotherapy (45 minutes a week during the inpatient and the outpatient program), crisis consultation if needed, and weekly meetings of the consultation team for all trainers and therapists for one hour. Staff also receives supervision twice-weekly.

Individual psychotherapy takes place on a weekly basis. The order of the topics of each session is based on Linehan’s protocol [16], and is pre-determined: suicidal and self-destructive behavior, therapy-interfering behavior, quality of life-interfering behavior and generalization of the skills taught in the training. Each therapy session starts by filling out diary cards that hold all the information concerning the problematic behaviors, which are the primary goal of the treatment, but also behaviors that influence the primary goals (such as alcohol and drug use, the urge to self-harm, substance abuse, dissociation, level of applied skills). Skills’ training takes place during the inpatient program on a weekly basis, but discussion of theory and homework classes are separated in time throughout the week (in total two and a half hours). The skills taught are standard DBT skills and combine self-regulation and change skills, and skills for self-acceptance and acceptance of others: Core mindfulness skills, Interpersonal effectiveness skills, Emotion regulation skills, Crises skills and Radical acceptance. Missed meetings need to be caught up by watching the video recordings that are made of all the trainings sessions.

Patients also receive daily mindfulness classes, two hours of drama therapy, psycho-educational classes about sexuality, substance abuse and medication, and the possibility of getting help in applying principles of validation and behavioral analysis skills. Other program parts are mainly based on living in a group, such as house-keeping meetings. In order to facilitate skills generalization, patients and staff developed psycho-educational evening sessions (two) in which information on BPD and DBT is given, followed by a training program (six sessions) in which patients together with family and friends can get help in applying skills. Each session lasts two hours. The first two sessions are spent on psycho-education about BPD, DBT and the content of the treatment program. In the next four sessions, all participants are asked to commit to practice the skills that are taught in the program with each other.

During evening and night hours, no staff is present. The results from the data gathered during the developmental phase of the program (October 2009 to September 2011), show that this does not lead to an increase in difficulties in handling relationships.

B) Standard outpatient DBT for 12 months

Patients assigned to standard dialectical behavior therapy receive 12 months of treatment as specified in the DBT manual [16] in one of the three participating regional psychiatric centers of Rivierduinen. The treatment is according to protocol and combines weekly individual cognitive-behavioral psychotherapy sessions with the primary therapist (45 minutes a week, weekly skills-training groups lasting two and a half hours per session (135 minutes), and if needed, consultation and weekly consultation meetings for trainers and therapists (one hour). Individual therapy, in both in- and outpatient programs, focuses primarily on motivational issues, including the motivation to stay alive and to stay in treatment. Group skills training [25] teaches Core Mindfulness skills, Interpersonal effectiveness skills, Emotion regulation skills, Crises skills and Radical acceptance.

### Selection of the therapists

Extensive attention was given to the selection of the therapists. Therapists and skills trainers per condition need to meet the highest quality requirements of good training, experience and supervision. All therapists/trainers are psychologists/psychiatrists, or registered nurses/social workers (n = 30). All have received at least a three-day introductory training in DBT principles administered by Dialexis, the training institute of the Dutch DBT association. Supervision will be given by the main researcher (LMCB), who is head of the Dutch DBT association and has received extensive training from Dr. Linehan in Seattle, USA. The treatment integrity in DBT is protected by systematic evaluation of video recorded individual and skill training sessions, by trained adherence coders.

### Objectives

The primary objective is to investigate whether this program is more effective in declining the proportion of patients that show parasuicidal behavior in the first three months of treatment, compared to standard outpatient DBT. We expect that after 12 weeks of inpatient DBT treatment, 20% of the patients will still show suicidal/self-harming behavior (measured by Life Time Parasuicide Count and items of the Borderline Personality Disorder Severity Index) compared to 60% of the patients in standard outpatient DBT. We expect this difference to be sustained after 24 weeks, though it may be reduced between 24 and 52 weeks.

The secondary objectives are: (i) to investigate whether short-term inpatient DBT is more effective in reducing other general (borderline) symptoms, (ii) to investigate whether short-term inpatient DBT is more effective in increasing quality of life, and (iii) to assess the cost-effectiveness of short-term inpatient DBT compared to outpatient DBT in terms of costs per reduced suicide attempts/self-destructive acts and cost per Quality Adjusted Life Year (QALY) from a societal perspective. We expect (i) a stronger decline in Borderline Personality Disorder Severity Index- and Brief Symptomatology Inventory scores and (ii) a stronger increase in quality of life (SF-36 and EQ-5D)-scores in the first three months of inpatient DBT as compared to the control group. We anticipate this difference to be sustained after 24 weeks, though it may reduce between 24 and 52 weeks. We also expect (iii) the initial higher cost of the intensive intervention will lead to better effects and possibly even lower cost than the control condition. Cost savings may be generated in the long run (after one year).

### Measurements

#### Screening

Vragenlijst voor Kenmerken van de Persoonlijkheid (VKP) [26], a paper-and-pencil self-report questionnaire that measures personality disorders as defined in the DSM-III-R [27] and the *International Classification of Diseases, version 10* (, ) [28], was used as a screening test. Next to a categorical diagnosis (‘negative’, ‘probable’ and ‘positive’), it yields a dimensional score for each disorder. Reliability and validity have proven to reasonable [29].

#### Axis I and axis II disorders

For inclusion, patients had to meet the criteria for borderline personality disorder, as measured by the Dutch translation of the *Structured Clinical Interview for DSM-IV, version II* (SCID-II) [30,31]. The SCID-II is a clinician-rated semi-structured clinical interview developed to measure the ten DSM-IV axis II personality disorders, supplemented by the depressive and the passive-aggressive personality disorder [32]. This instrument has shown adequate interrater and internal consistency reliability [33].

The web-based Dutch version of the *Mini International Neuropsychiatric Interview* (MINI-PLUS) [34,35] was used to assess exclusion criteria. The MINI-PLUS is a structured clinician-rated diagnostic interview that is used to determine the most common DSM-IV [32] and ICD-10 psychiatric disorders [28]. Reliability and validity are considered good [34,36].

The interviewers who conducted the SCID-II and MINI-PLUS interviews had a master’s degree in psychology. They received training in how to carry out the interviews and were supervised by an experienced clinical psychologist. The interviewers had the opportunity to verify their diagnosis with a psychiatrist.

### Outcomes

*Frequency of suicide attempts/self-harming acts*, as measured by the *Lifetime Parasuicide Count* (LPC: [37]. The LPC is a sixteen-item clinician-rated interview that determines parasuicide markers (date, method, intent, and medical treatment) of respectively the first, most recent and most severe parasuicide, and determines the frequency and subsequent medical treatment of 12 methods of self-mutilating behaviors (for example, cutting, burning and pricking). Data on the psychometric properties of this instrument were not available.

The *Borderline Personality Disorder Severity Index* (BPDSI) [38] is a clinician-rated semi-structured interview assessing the frequency of borderline symptoms in the previous three-month period. The BPDSI-IV has shown high interrater reliability, moderate to high internal consistencies and very good discriminant, concurrent and construct validity [38,39]. The BPDSI-IV has been used in various trials and proved to be sensitive to change among others. [40,41]. Patients have reported that during the interview they feel their problems are acknowledged [42].

The *SF-36*[43], or *Medical Outcomes Study 36-Item Short Form Health Survey*, is a short-form, self-report health survey with 36 questions. It yields an eight-scale profile of functional health and well-being: Physical Functioning, Role-Physical, Bodily Pain, General Health, Vitality, Social Functioning, Role-Emotional and Mental Health as well as psychometrically-based physical and mental health summary measures and a preference-based health utility index. A higher score indicates a better state of health [43]. A Dutch version was translated and validated by Aaronson *et al*. [44]. Data from the SF-36 were collected using a computer-based version, *SF-6D.*

*SF-6D.* The Sheffield Health Economic Group derived a preference-based measure of health from the SF-36 [45]. The new preference-based measure, known as the SF-6D, is derived from 11 items of the SF-36 and is composed of six dimensions of health with four to six levels each.

The *EQ-5D*[46], or *EuroQol 5 Dimensions* descriptive system, is a paper-and-pencil self-report questionnaire that consists of five dimensions (Mobility, Self Care, Usual Activities, Pain/Discomfort and Anxiety/Depression) with three levels each (no problems, some problems and extreme problems), thus defining 243 distinct health states. Each of these health states can be assigned a particular utility using a scoring algorithm. The Dutch tariffs were determined in a time trade-off study. EQ-5D utilities range from −0.329 to 1. Assessment takes one to two minutes.

The *TiC-P* or ‘*Trimbos and iMTA Questionnaire on Costs Associated with Psychiatric Illness*’ (TiC-P) [47] is a validated tool commonly applied in economic evaluations of treatments in mental health care. The TiC-p is a paper-and-pencil self-report questionnaire that consists of two parts. The first part measures direct medical costs. The second part estimates the productivity costs. It includes a short form of the Health and Labor questionnaire (HLQ), consisting of three modules that measure productivity losses: absence from work, reduced efficiency at work and difficulties with job performance [48]. The TIC-P is considered to be a feasible and reliable instrument for collecting data on medical consumption and productivity losses in patients with mental health problems. The construct validity of questions related to contacts with psychotherapist and long-term absence from work is satisfactory [49].

The BSI, or *Brief Symptomatology Inventory* (BSI) [50], is a self-report questionnaire that includes 49 items grouped into nine scales that encompass nine primary dimensions of psychopathological symptoms: psychoticism, somatization, depression, hostility, phobic anxiety, obsessive-compulsivity, anxiety (panic), paranoid ideation, and nervous tension. Derogatis and Melisaratos [50] presented appropriate coefficients of internal consistency of the BSI ranging from 0.71 to 0.85, test-retest reliability coefficients ranging from 0.68 to 0.91, and evidence for the a good construct validity of the BSI. Other studies have reported similar estimates, for example, see references [51,52]. Data of the BSI were collected using a computer-based version.

### Sample size

We expect that after 12 weeks, 20% of the patients of the intervention group will still show suicidal and/or self-harming behavior compared to 60% of the patients in the control condition. To be able to detect a difference of 40% after 12 weeks, with a power of 0.80 and an α = 0.05, a minimum of 23 patients per experimental condition is required. We will randomize between approximately 70 patients during the study period to guarantee enough power, taking into account an expected 30% drop-out rate.

### Randomization

#### Sequence generation

Treatment allocation is carried out by a computer program, developed by the Amsterdam Medical Center. To increase the likelihood of comparable treatment groups, a minimization method is preferred. To preserve the random character of allocation, a biased coin procedure will be used. In case of imbalance, the allocation probability is modified from 50/50 to 80/20. Minimization variables are: level of severity of borderline symptomatology (BPDSI score ≥ 40), level of suicidality/self-harming behavior (LPC score ≥ 14), and age.

#### Allocation concealment

The sequence was concealed until interventions were assigned.

### Implementation

When patients register in the participating psychiatric centers, crisis team members or staff members working at the front office decide whether or not this patient is a candidate for DBT, based on a rapid assessment of BPD symptomatology. Patients are informed about the central recruitment and diagnostic facility and are given the phone numbers of the project leader and coordinator. After a short, standardized telephone interview with the project leader or the coordinator, in which general information on the interventions and study is given, patients need to fill out a screening instrument (Vragenlijst Kenmerken Persoonlijkheid, VKP) [26] and send it back to the central diagnostic facility. Subsequently, a psychologist contacts the patient for a briefing of the results. If the screening instrument indicates that presence of BPD is probable, then psychologist and patient make an appointment for a thorough examination within three weeks. During this thorough examination, with use of the MINI-PLUS and SCID-II, presence of DSM axis I and axis II disorders are verified. Frequency of borderline complaints and parasuicidal gestures are assessed using respectively BPDSI and LPC. All assessment tools employed have demonstrated reliability and validity (described in detail below). Informed consent is discussed and signed. After signing the informed consent, patients are randomized and the diagnostician reports the allocation of the treatment back to the patients. The allocated treatment center then contacts the patient and starts treatment.

### Blinding

No blinding took place.

### Statistical analysis

All participants who are randomised will be included in the comparison and analyzed according to their randomized allocation (intent-to-treat analysis). Wherever possible, we will continue to collect follow-up data from participants after they drop out of treatment, so that the dataset will be as complete as possible. All analyses will be carried out using SPSS (Statistical Package for the Social Sciences) 19 [53]. The effect of the intervention in terms of proportion of respondents still showing suicidal and/or self-destructive behavior after 12 weeks, and the retention of this effect between 24, 36 and 52 weeks, will be computed with the help of longitudinal logistic regression, with time, experimental condition and their interaction as independent variables. The effect of the intervention in terms of reduction in mean BPDSI, LPC, SF-36, and the BSI ratings over time, will be computed with the help of a mixed model linear regression analysis with time, experimental condition and their interaction as independent variables. Missing values will be taken into account by using full information maximum likelihood method estimation. Since there may be some heterogeneity in the implementation of standard DBT across the three different regional centers, between-center heterogeneity will be explored in subgroup analyses.

### Cost-effectiveness analysis

The economic evaluation will be undertaken from a societal perspective. Hence, all relevant effects and costs due to resource utilization within healthcare (direct medical costs) and productivity costs will be included. To examine the cost-effectiveness of the inpatient program compared to the outpatient program the EQ-5D, the SF-6D, and the TiC-P, will be used. We will assess the cost-effectiveness of short-term inpatient DBT compared to outpatient DBT in terms of costs per reduced suicide attempts/self-destructive acts and cost per QALY, the so-called cost utility analysis. The cost utility will be evaluated by relating the difference in direct medical costs per patient receiving inpatient treatment and outpatient care to the difference in terms of QALYs, which yields a cost per QALY estimate. We will estimate the cost per QALY including productivity costs.

The uncertainty will be assessed using bootstrapping, and the results will be presented in acceptability curves.

### Ethical precautions and crisis management

The study will be executed in accordance with the guidelines for Good Clinical Practice, the principles of the Declaration of Helsinki, the Medical Research Involving Human Subjects Act (WMO) and other guidelines, regulations and Acts as currently used in GGZ Rivierduinen. Ethical approval is obtained from the ethical review board of the Leiden University Medical center. The board of Rivierduinen agreed to support the execution of the study. All the boards of the psychiatric regional centers that take part in the study also gave agreement. All participants will be extensively informed about the study, addressing confidentiality, the right to abort their participation at any time and without clarification. Patients can leave the research program any time they want, and this will not affect the course of their treatment. Written information is given. When the patient is willing to continue, written consent is asked. A computer program, developed by the Amsterdam Medical Center, carries out treatment allocation. This program rules out determination of group assignment, which guarantees impartiality of the researchers. The diagnostics and follow-up measurements take place in the Jelgersma Center. An independent physician is appointed, to whom subjects can address questions about the research before, during and after a study. The independent physician does not work at the institution where the study is being carried out, and is not involved in the study itself. Patients will receive a voucher of 10 euros for each repeated measurement, and restitution of their travel expenses.

## Discussion

Mental health care professionals are frequently confronted by a dilemma when treating highly suicidal patients in an outpatient setting. Hospitalization seems inevitable under some circumstances, yet has proven harmful in its own right. This paper outlines the background and methods of a randomized trial evaluating the possible surplus value of a short-term inpatient DBT program.

Although previous quasi-experimental studies [22,23] have shown positive effects of inpatient DBT, the setting could add to the risk of regressive processes during treatment. Therefore we evaluated outcomes of the experimental short-term inpatient DBT program of the Jelgersma Center in a quasi-experimental study, comparing pre- and post-intervention data for 39 female patients with a diagnosis of BPD [54]. It was found that the severity of borderline problems, particularly in the field of interpersonal problems, was significantly reduced. In general, suicidality and parasuicidal behavior were not reduced significantly, though positive effects for patients reporting high levels of suicide attempts and self-harming behavior at baseline were found. It was concluded that the experimental program showed promise for patients who show high levels of parasuicidal behavior and that further development and research was justified. Improvements were suggested and implemented [54]. One of the most important changes made was restricting the program to patients with recent suicide attempts and severe self-harming behavior.

Four centers of GGZ Rivierduinen (Center of Personality Disorders Jelgersma, GGZ Rijnstreek, GGZ Leiden en GGZ Midden-Holland) agreed to participate in the trial. In order to rule out differences in the admittance process, an independent, central diagnostic facility was established. This resulted in an efficient, standardized intake procedure that bypasses the waiting lists in the centers. This adaption was considered to be beneficial for participants, since they know their diagnosis and the treatment program they will be part of within a few weeks.

However, problems can be expected as a result of the randomization. The fact that allocation of setting is random (outpatient versus inpatient) will create an important toll for participants, because they may be randomized to a treatment condition that was not their primary choice. This involves acceptance of uncertainty. Furthermore, allocation to the experimental program involves an inpatient setting for 12 weeks, which may be difficult to organize for patients with families and work.

Another important problem results from the fact that although an independent, central diagnostic facility was created, the study has no influence on the speed with which patients enter the treatment conditions. The outpatient DBT teams that are part of the trial have a limited capacity and as a result usually have waiting lists.

## Trial status

Recruiting

## Abbreviations

BPD: Borderline Personality Disorder; BPDSI: Borderline Personality Disorder Severity Index; BSI: Brief Symptomatology Inventory; DBT: Dialectical Behavior Therapy; DSM-IV: *Diagnostic and Statistical Manual of Mental Disorders*, *version IV*; EQ-5D: EuroQol 5 Dimensions; HLQ: Health and Labor questionnaire; ICD-10: *International Classification of Diseases, version 10*; LPC: Lifetime Parasuicide Count; MINI-PLUS: A web-based Dutch version of the *Mini International Neuropsychiatric Interview*; QALY: Quality Adjusted Life Year; SF-36: Medical Outcomes Study 36-Item Short Form Health Survey; SF-6D: Six dimensions of health derived from the SF-36; TiC-P: Trimbos and iMTA Questionnaire on Costs associated with Psychiatric illness.

## Competing interests

The authors declare that they have no competing interests.

## Authors’ contributions

LvdB: conception and design, drafting the work or revising it critically, data collection and analysis, manuscript writing and final approval of the manuscript. RS: substantial contributions to the design of the work, data collection and analysis, critical revision and final approval of the manuscript. LH: substantial contributions to the design of the work, data collection and analysis, revising it critically for important intellectual content and final approval of the manuscript. EvF: financial support, substantial contributions to the acquisition of data for the work and final approval of manuscript. Drafting the work or revising it critically for important intellectual content. All authors read and approved the final manuscript.
